# Immunometabolism of regulatory T cells in cancer

**DOI:** 10.1038/s41388-025-03458-1

**Published:** 2025-06-04

**Authors:** Jordy Saravia, Hongbo Chi

**Affiliations:** Department of Immunology, St. Jude Children’s Research Hospital, Memphis, TN USA

**Keywords:** Lymphocytes, Immunosurveillance

## Abstract

Regulatory T (T_reg_) cells play critical roles in maintaining immune tolerance and tissue homeostasis, but impede anti-tumor immunity. Recent work has established how T_reg_ cells metabolically adapt within the tumor microenvironment (TME), and these adaptations frequently provide a functional advantage over effector T cells. Further, enhanced T_reg_ cell function in the TME may contribute to the limited efficacy of current immunotherapies, especially immune checkpoint blockade (ICB). Here, we review recent progress in understanding mechanisms of T_reg_ cell heterogeneity and function in tumors, with a particular focus on cellular metabolism as an underlying factor by which T_reg_ cells are uniquely poised to thrive in the TME and contribute to tumorigenesis. We describe how cellular metabolism and nutrient or metabolic communication shape T_reg_ cell lineage identity and function in the TME. We also discuss the interplay between ICB and T_reg_ cell metabolism and function, and highlight current strategies targeting T_reg_ cell metabolism specifically in the TME. Understanding metabolic control of intratumoral T_reg_ cells provides excellent opportunities to uncover new or combination therapies for cancer.

## Introduction

Immunotherapies such as immune checkpoint blockade (ICB) or adoptive cell therapy (ACT) show great potential as effective treatments for cancer. However, the therapeutic efficacies of these treatments are often limited due to multiple factors, including poor infiltration or persistence of effector cell populations or their reprogramming into dysfunctional or immunosuppressive states in the tumor microenvironment (TME). The TME is a complex mixture of cell types, including tumor and immune cells, and this cellular milieu is associated with an altered metabolic state of malignant cells. Specifically, tumor cells acquire and consume high levels of nutrients and produce immunosuppressive metabolites to support their growth and proliferation. In turn, these changes create a condition of metabolic competition or stress wherein the anti-tumor functions of macrophages, dendritic cells (DCs), NK cells, and conventional CD4^+^ and CD8^+^ T cells are reduced as recently summarized elsewhere [1–5]. Therefore, metabolic alterations in tumor cells have emerged as a hallmark of cancer [6].

CD4^+^ regulatory T (T_reg_) cells play a major role in limiting anti-tumor immunity, and T_reg_ cell accumulation in tumors is often negatively associated with clinical outcomes and immunotherapeutic efficacy [7, 8]. While strategies that promote T_reg_ cell depletion or dysfunction may overcome the obstacles with immunotherapeutic efficacy, these strategies can be associated with the development of autoimmune disorders owing to the requirement of T_reg_ cells for mediating immunosuppression under homeostasis. Thus, it is critical to determine specific approaches to limit T_reg_ cell immunoregulatory activity in the TME while minimizing the systemic impacts on disrupting immune tolerance or tissue homeostasis. In contrast to those immune cell types that restrict tumor growth, emerging studies establish that T_reg_ cells often undergo metabolic adaptation to maintain immunosuppressive function in the TME. In this review, we discuss mechanisms underlying T_reg_ cell immunosuppressive function within the TME, with a focus on cellular metabolism as a primary factor underlying the ability of T_reg_ cells to thrive in the TME. First, we discuss mechanisms of T_reg_ cell immunosuppressive function in the TME. Then, we describe how cellular metabolism and nutrient or metabolic communication shapes T_reg_ cell stability and function in the TME and the consequences on anti-tumor immunity. Given their high expression of immune checkpoint molecules, we discuss how altering T_reg_ cell metabolism impacts ICB efficacy, and conversely, how ICB affects T_reg_ cell metabolic function. Finally, we summarize our current understanding of the specific molecular and metabolic processes underlying intratumoral T_reg_ cell function.

## Malicious compliance: T_reg_ cell guardianship of “self” at the cost of tumorigenesis

T_reg_ cells are a functionally and metabolically unique arm of the adaptive immune system that is responsible for maintaining immune homeostasis and tissue tolerance. Whereas pro-inflammatory functions of conventional T cells help eliminate invading pathogens or tumor cells, T_reg_ cells suppress pro-inflammatory immune cell activation via multiple mechanisms [9]. T_reg_ cells differentiate either directly from thymic precursors (called tT_reg_ cells) or from naïve CD4^+^ T cells in peripheral tissues (called pT_reg_ cells) including tumors; further, they are defined by expression of the master transcription factor FOXP3 [10] that controls the expression of many factors critical for T_reg_ cell immunosuppressive function such as CTLA4 and ICOS [11]. Indeed, mice lacking functional FOXP3 from birth [12, 13] or adult mice undergoing T_reg_ cell ablation [14] develop a fatal lymphoproliferative disease due to aberrant activation of T cells. This disease effect is reversed by genetically reinstating FOXP3 expression in CD4^+^ T cells [15], demonstrating that T_reg_ cells are both necessary and sufficient to maintain immune homeostasis. Finally, T_reg_ cells are highly heterogeneous and display differential gene expression and epigenetic profiles, corresponding to their specific activation state or tissue location [16–18].

**Fig. 1:**
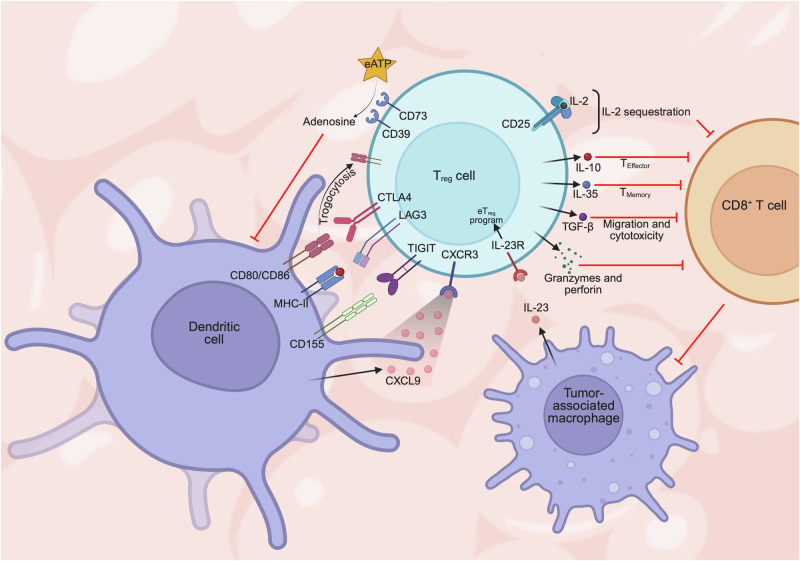
Mechanisms of T_reg_ cell-mediated suppression in tumors. T_reg_ cells mediate immunosuppression in the tumor microenvironment (TME) through various cell contact-dependent and contact-independent mechanisms. T_reg_ cells migrate in proximity to dendritic cells (DCs) in the TME via the CXCL9–CXCR3 chemokine chemokine receptor axis. Molecules expressed on the T_reg_ cell surface, such as CTLA4, LAG3, and TIGIT, bind to CD80/CD86, MHC-II, and CD155, respectively, on antigen presenting cells (APCs) such as DCs, thereby limiting the availability of these stimulatory signals for conventional T cells (e.g., CD8^+^ T cells). T_reg_ cells sequester IL-2 via expression of the high-affinity IL-2 receptor CD25. T_reg_ cell-derived immunosuppressive cytokines limit the development of effector T cells (by IL-10), memory T cells (by IL-35), and also the migratory and cytotoxic capacity of CD8^+^ T cells (by TGF-β). T_reg_ cells produce granzymes and perforin for cytotoxic and cytolytic effects on CD8^+^ T cells. Extracellular ATP (eATP) is enzymatically converted to adenosine by the CD39 and CD73 ectoenzymes expressed on T_reg_ cells, resulting in suppression of anti-tumor immunity. Tumor-associated macrophages (TAMs) produce IL-23 in the TME, which maintains highly suppressive effector T_reg_ (eT_reg_) cells in the TME. In turn, T_reg_ cells indirectly support TAM phenotypes via suppression of CD8^+^ T cells.

Though T_reg_ cells are indispensable for immune tolerance and the prevention of autoimmunity, their suppressive function is detrimental to anti-tumor immunity. Since tumor cells originate from “self” tissues, tumors may exploit the self-antigen-sensing function of T_reg_ cells. For example, self-antigen-specific (albeit tumor-non-reactive) T_reg_ cells are enriched in prostate tumors [19]. In addition, TCR repertoire analyses have revealed considerable heterogeneity among intratumoral T_reg_ cells, including those that are specific for tumor-associated antigens or neoantigens [20] or those that have differentiated from previously activated conventional CD4^+^ T cells [21, 22]. T_reg_ cell accumulation and/or increased ratio of T_reg_ cells:CD8^+^ T cells in tumors is often negatively associated with patient prognosis and survival [23, 24], including in ovarian [25, 26], breast [27, 28], lung [29, 30], and liver [31, 32] cancers. Further, T_reg_ cells are abundant within the TME of “hot” tumors [25–32] and limit the pro-inflammatory functions of innate and adaptive immune cells via multiple mechanisms as described below (Fig. 1).

Conventional T cells are major mediators of anti-tumor immunity and require antigen (via TCR recognition; signal 1), co-stimulatory (signal 2), cytokine (signal 3), and nutrient (e.g., glucose, amino acids, lipids; signal 4) signals for their activation and effector differentiation [33, 34]. T_reg_ cells can disrupt co-stimulatory signals between antigen-presenting cells (APCs). For example, T_reg_ cells impede DC–T cell interactions via expression of CTLA4, which competes with CD28 co-stimulatory receptor (expressed on T cells) for binding to CD80 or CD86 (expressed on adjacent DCs), culminating in decreased T cell activation. CTLA4^+^ T_reg_ cells also directly extract CD80 and CD86 co-stimulatory molecules from APCs via trogocytosis [35], and intratumoral DCs show increased expression of these molecules upon depletion of intratumoral T_reg_ cells [36]. Intratumoral T_reg_ cells also highly express the checkpoint molecule LAG3 [37, 38], which competes for MHC-II binding with TCR and reduces expression of co-stimulatory molecules on APCs [38]. Additionally, TIGIT^+^ T_reg_ cells enforce the tolerogenic function of DCs, owing to the competitive binding of TIGIT to the co-stimulatory molecule CD155, thereby limiting its engagement of CD226 expressed by T cells [39].

Intratumoral T_reg_ cells also disrupt APC co-stimulation by exploiting CXCL9–CXCR3 chemokine signaling, resulting in DCs preferentially interacting with T_reg_ cells over CD8^+^ T cells [40]. Another critical immunosuppressive mechanism of T_reg_ cells is IL-2 sequestration. Specifically, T_reg_ cells highly express IL-2Rα (CD25) [41], which has a much higher affinity for IL-2 than IL-2Rβ (CD122) chain expressed by conventional T cells [42], thereby decreasing IL-2 availability for nearby conventional T cells. The combined restriction of signals 2 and 3 consequently impedes the survival, differentiation, and anti-tumor function of T cells. Interestingly, reinvigoration of CD8^+^ T cells by anti-PD-1 ICB increases IL-2 production, which in turn increases ICOS expression on T_reg_ cells to increase their stability and accumulation in tumors. Such effects dampen the efficacy of anti-PD-1 therapy and can be blocked by pre-treatment with anti-ICOSL antibody [43]. Of note, the cytokine IL-23 is produced by tumor-associated macrophages (TAMs) and maintains a highly suppressive effector T_reg_ (eT_reg_) cell phenotype in tumors [44], highlighting complex cell–cell communication between T_reg_ cells and other immune cells in the TME.

T_reg_ cells also utilize cell contact-independent mechanisms to suppress immune cell functions. The immunosuppressive cytokines IL-10 and IL-35 are expressed by distinct intratumoral T_reg_ cell populations, and such heterogeneity enables the suppression of both effector (via IL-10) [45, 46] and memory (via IL-35) [45–47] T cell responses. TGF-β is another immunosuppressive cytokine produced by T_reg_ cells, leading to defects in CD8^+^ T cell cytotoxic function and trafficking to tumors [48, 49]. Intratumoral T_reg_ cells may also express the surface ectoenzymes CD39 and CD73 that convert extracellular ATP to adenosine, a metabolite with immunosuppressive effects as described in more detail later in this review [50, 51]. Finally, T_reg_ cells can also produce granzymes and perforin to exert direct cytotoxic function against pro-inflammatory immune cells, which impacts anti-tumor immune cells [52]. Taken together, T_reg_ cells deploy multiple strategies to suppress anti-tumor immunity, and the functional redundancies and/or adaptation between these mechanisms likely underlie why direct targeting of T_reg_ cell suppressive functions is challenging.

## Tumors as a favorable metabolic niche for T_reg_ cells

**Fig. 2:**
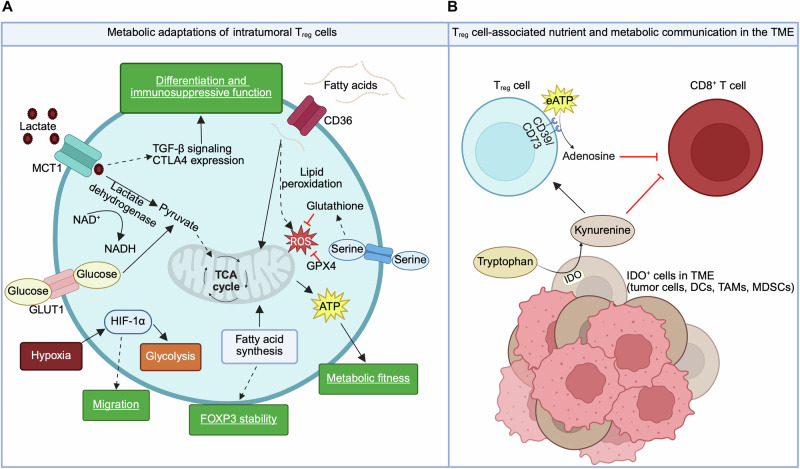
Tumors as a favorable metabolic niche for T_reg_ cells. **A** T_reg_ cells uptake available lactate in the TME via the lactate transporter MCT1. Lactate is converted to pyruvate via lactate dehydrogenase and then shuttled into the TCA cycle to support cellular ATP production and overall metabolic fitness. Increased intracellular lactate also promotes T_reg_ cell differentiation and immunosuppressive function by enhancing TGF-β signaling and CTLA4 expression. Likewise, glucose uptake via its transporter GLUT1 is another mechanism for cellular energy production. Intratumoral T_reg_ cells rely on fatty acid metabolism for energy production and FOXP3 stability, including de novo fatty acid synthesis and uptake of exogenous fatty acids via CD36. Increased expression of antioxidant mechanisms such as GPX4 and serine-derived glutathione production helps to shield T_reg_ cells from ROS-induced damage that is associated with increased fatty acid oxidation. Hypoxic conditions in the TME activate HIF-1α, which boosts glycolytic metabolism in T_reg_ cells and promotes T_reg_ cell migration into tumors (see text for more details). **B** Extracellular adenosine levels are increased in the TME, owing to its conversion from eATP via CD39 and CD73 expressed on T_reg_ cells. Adenosine has immunosuppressive effects on CD8^+^ T cells and certain antigen-presenting cell populations (not depicted). Tumor and myeloid cells (e.g., myeloid-derived suppressor cells (MDSCs)) in the TME express indoleamine-2,3-dioxygenase (IDO), which metabolizes tryptophan to kynurenine. Kynurenine promotes T_reg_ cell differentiation and function, which inhibits CD8^+^ T cell function.

Cellular metabolism has emerged as a function-defining feature of immune cells and putative therapeutic target. T_reg_ cells are metabolically distinguished from conventional T cells. As compared with effector CD4^+^ T cells, T_reg_ cells are less dependent on glycolysis and instead rely upon mitochondrial oxidative phosphorylation (OXPHOS) to produce energy [53–55], and these metabolic characteristics are regulated by FOXP3 [56]. Accordingly, mitochondrial metabolism plays a critical role in orchestrating T_reg_ cell survival and suppressive function in vivo [57–60]. Mechanistically, mTOR- and MYC-dependent signaling drive metabolic reprogramming in T_reg_ cells to support mitochondrial biogenesis and fitness, as well as lipid biosynthesis and downstream post-translational modifications; these metabolic pathways and signaling processes dictate T_reg_ cell activation and suppressive function [57, 58, 61–63]. Recent studies also identified nutrient transport, sensing, and signaling mechanisms as crucial upstream signals mediating mTORC1 activation in T_reg_ cells [64, 65]. Nonetheless, mTOR-associated signaling pathways must be carefully balanced to provide enough energy for proliferation and suppressive function without losing FOXP3 stability and T_reg_ cell identity. For example, mice with T_reg_ cell-specific deletion of mTOR [57] or the obligate mTORC1 complex molecule Raptor (but not the obligate mTORC2 complex molecule Rictor) [62] develop a severe, fatal autoimmune disease. However, mice with uncontrolled mTORC1/2 signaling in T_reg_ cells, such as due to T_reg_ cell-specific deletion of ATG7 [66] (mediated by mTORC1 activation) or PTEN [67, 68] (caused by increased mTORC2 function), or mice with T_reg_ cell-specific overexpression of glucose transporter GLUT1 [56] also develop autoimmune symptoms due to decreased FOXP3 stability and T_reg_ cell function; all of these phenotypes are associated with aberrant glycolysis [56, 66–68]. Thus, there is a “Goldilocks” effect of mTOR signaling and metabolic programs for tuning T_reg_ cell function [69]. It is becoming more appreciated that T_reg_ cells are metabolically heterogeneous [70–76]. Emerging studies highlight that the nutrient and metabolic requirements of different T_reg_ cell states are shaped by immune and tissue contexts [77–79], including in tumors, and that the capacity to metabolically adapt to tissue niches underlies a functional advantage of T_reg_ cells to thrive within tumors (Fig. 2). In this section, we discuss how specific features of the TME promote immunosuppression, and the metabolic mechanisms involved in enhancing T_reg_ cell accumulation and function to limit anti-tumor immunity.

In the TME, immune cells must adapt to nutrient scarcity, hypoxia, and low pH conditions [2]. One hallmark of malignant cell transformation is the switch to glycolytic catabolism to support aberrant cellular proliferation [2]. This process is associated with enhanced cellular uptake of nutrients such as glucose and amino acids. Consequently, the finite availability of these in-demand nutrients, combined with an abundance of lactate produced by tumor cells, leads to suboptimal microenvironmental conditions to support anti-tumor immune cell function and survival. As opposed to conventional CD4^+^ and CD8^+^ T cells, T_reg_ cells more readily metabolically adapt to the TME, and this metabolic plasticity selectively sustains their accumulation and immunosuppressive effects as discussed below (Fig. 2A). For example, intratumoral T_reg_ cells utilize extracellular lactate by increasing expression of its transporter monocarboxylate transporter-1 (MCT1) and the enzyme lactate dehydrogenase, which shuttles lactate into the TCA cycle (via conversion to pyruvate) to support OXPHOS [80, 81]. Further, this mechanism effectively replenishes NAD^+^ stores in T_reg_ cells but not in conventional CD4^+^ and CD8^+^ T cells that oxidize excessive lactate, thereby limiting NAD^+^-dependent support of activation-associated glycolytic metabolism [82]. Elevated intracellular lactate also enhances OXPHOS in T_reg_ cells by promoting expression of acetylglucosaminyltransferase, which is important for post-translational modification of mitochondrial proteins [83]. In addition to metabolic reprogramming effects, lactate promotes T_reg_ cell stability by enhancing TGF-β signaling through SMAD3 [84]. Additionally, lactate uptake by T_reg_ cells regulates RNA splicing machinery in intratumoral T_reg_ cells; in turn, CTLA4 expression is induced, thereby promoting the efficacy of anti-CTLA4 therapy [85]. Further, anti-CTLA4 promotes metabolic rewiring in T_reg_ cells to further destabilize their pro-tumorigenic function in glucose-deprived TME [86]. Interestingly, lactate causes a similar anti-inflammatory effect in TAMs, promoting an M2-like phenotype associated with decreased tumor control [87], which may also affect T_reg_ cell–TAM interactions (i.e., via TAM-derived IL-23) in the TME [44]. Together, these studies demonstrate the multi-potent effects of lactate in negatively regulating pro-inflammatory gene programs in favor of immunosuppressive gene programs in the TME.

From a therapeutic perspective, limiting lactate production by tumor cells can decrease intratumoral T_reg_ cell accumulation and function, as demonstrated in one study via treatment with the curcumin analog GO-Y030 [88]. These effects may be mediated by direct metabolic or signaling effects of lactate, as discussed above, or alterations of acidity in the TME. Indeed, tumor-derived lactate, together with elevated local CO_2_ due to hypoperfusion, can lower the pH of the TME, and there is emerging evidence that acidity promotes T_reg_ cell differentiation and immunosuppressive function independently of lactate. Specifically, the enhanced differentiation of T_reg_ cells upon treatment with lactate (pH 6.8) is abrogated with pH-neutral sodium lactate but recapitulated under HCl-acidified media conditions [89]. Additionally, T_reg_ cell pretreatment with acidified media appears to increase their suppressive function in an adoptive transfer model, associated with increased tumor growth and impaired intratumoral CD8^+^ T cell infiltration [90]. Thus, more work is warranted to better understand the overlapping and distinct effects of lactate versus general acidity on intratumoral T_reg_ cells.

Tumor cells must increase lipid synthesis to meet biosynthetic demands of proliferation, and also use lipids as an energy source for OXPHOS via fatty acid oxidation. As such, lipids are often plentiful in tumors, and alterations in lipid metabolism are linked to tumor progression and resistance to immunotherapies [91]. Similar to their adaptive utilization of lactate, T_reg_ cells upregulate lipid metabolism pathways to support their survival and immunosuppressive functions in tumors [92–94]. Indeed, blockade of free fatty acid release by tumor cells or free fatty acid uptake by T_reg_ cells (via anti-CD36 antibody treatment or T_reg_ cell-specific deletion of CD36) decreases intratumoral T_reg_ cell accumulation and reverses tumor resistance to anti-PD-1 treatment [93, 95]. Importantly, the adaptive capability to increase lipid storage is critical to maintain intratumoral T_reg_ cell identity [96]. In addition to uptake of exogenous free fatty acids, intratumoral T_reg_ cells increase de novo synthesis of fatty acids via activation of SREBPs and downstream fatty acid synthase (FASN), which supports T_reg_ cell suppressive function by protecting against TCR signaling-mediated T_reg_ cell fragility [92, 94]. Deficiency of SCAP (an obligatory regulator of SREBPs) or FASN in T_reg_ cells inhibits tumor growth without systemic autoimmunity toxicity [92]. Of note, inhibition of FABP5 (a fatty acid binding protein) in T_reg_ cells disrupts intracellular lipid trafficking and mitochondrial metabolic fitness, which increases the suppressive function of T_reg_ cells [97]. Thus, deeper mechanistic dissection of distinct components involved in T_reg_ cell lipid uptake, intracellular transport, and de novo synthesis is warranted to unravel the mechanisms underlying the complexities of lipid metabolism in intratumoral T_reg_ cells.

In line with this notion, the sphingolipid intermediate sphinganine interacts with the transcription factor c-FOS and enhances its recruitment to target genes such as *Pdcd1* (encodes PD-1) [98]. The peroxisome proliferator-activated receptor (PPAR) transcription factors, which are activated by certain lipids, also contribute to T_reg_ cell biology. For example, PPAR-γ plays critical roles in T_reg_ cell programming and accumulation in multiple non-lymphoid tissues [16, 99]. Additionally, PPAR-β functions downstream of CD36 to support lipid-associated metabolic adaptation of intratumoral T_reg_ cells [93], thereby linking lipid metabolism to transcriptional regulation of T_reg_ cells in tumors. Finally, steroid hormones such as glucocorticoids have well-known immunosuppressive effects, and it was recently shown that some tumors can enzymatically regenerate glucocorticoids from inactive metabolites, thereby dampening local immune responses [100]. Intratumoral T_reg_ cells exposed to tumor-derived glucocorticoids showed enriched T_reg_ cell activation gene signatures [100]. The pleotropic effects of glucocorticoids on T_reg_ cells and non-T_reg_ immune cells suggest a two-pronged mechanism for tumor cells to evade anti-tumor immunity.

Increased lipid catabolism is associated with the production of cellular ROS. Although intratumoral CD8^+^ T cells may also increase lipid uptake via upregulation of CD36, increased lipid peroxidation and ROS production in those cells induce ferroptotic cell death and ultimately dampen anti-tumor cytokine production [101, 102]. Further, T_reg_ cells can be made susceptible to lipid peroxidation-induced ferroptosis via deletion of the enzyme glutathione peroxidase 4 (GPX4) without affecting T_reg_ cell homeostasis in other tissues [103], suggesting that redox balance dictates intratumoral T_reg_ cell function. Accordingly, T_reg_ cells also synthesize the critical antioxidant molecule glutathione (GSH), and disruption of serine-dependent GSH generation in T_reg_ cells results in boosting of anti-tumor immunity, albeit at the expense of developing autoimmunity [104]. However, these autoimmune effects are rectified by feeding mice bearing GSH-deficient T_reg_ cells a serine- and glycine-deficient diet [104], which is interesting considering that a serine-free diet also limits intratumoral T_reg_ cell function and inhibits tumor growth [98]. Thus, intratumoral T_reg_ cells prioritize the control of ROS, which represents a putative target to dampen T_reg_ cell function in tumors.

Owing to limited vascularity, solid tumors often contain hypoxic regions that are conducive for the hypoxia-inducible factor 1α (HIF-1α) activation, thereby reprogramming glucose metabolism towards the generation of lactate over pyruvate [60]. HIF-1α negatively regulates the differentiation of T_reg_ cells in vitro by directly inhibiting FOXP3 [54, 105, 106]. However, HIF-1α enhances T_reg_ cell differentiation and suppressive function under hypoxic conditions in vivo, such as in colon cancer [107] and inflammation [108]. Further, hypoxia promotes recruitment of T_reg_ cells to tumors and helps trigger vascular endothelial growth factor (VEGF) production for the expansion of intratumoral T_reg_ cells [109, 110]. Similarly, hypoxia and HIF-1α support T_reg_ cell migration into glioblastoma tumors, although T_reg_ cell suppressive function is decreased under such conditions due to aberrantly increased glycolysis [60]. Accordingly, HIF-1α-deficiency boosts T_reg_ cell suppressive function in hypoxic glioblastoma tumors [60], possibly due to their preferences for lactate or lipid metabolism as discussed above [81, 92–94].

Additionally, increased glycolysis may enhance the immunosuppressive function of activated T_reg_ cells in humans [111], and thus, the specific roles of HIF-1α in different clinical contexts remain to be elucidated. Interestingly, excessive availability of the tumor-derived oncometabolite D-2-hydroxyglutarate destabilizes HIF-1α and boosts OXPHOS in T_reg_ cells, thereby promoting their accumulation in tumors [112]. Besides glycolysis, HIF-1α can induce autophagy under hypoxic conditions [113]. Further, autophagy is required for T_reg_ cell stability and survival [66, 114], as well as their function to suppress anti-tumor immunity and autoimmunity [66]. Beyond T_reg_ cells, hypoxia-associated HIF-1α activation also promotes immunosuppressive phenotypes in tumor-resident γδ T cells [115] and TAMs [116], highlighting a broad effect of hypoxia in limiting anti-tumor immunity.

T_reg_ cell differentiation and function are regulated by nutrient and metabolite communication with neighboring cells in the TME (Fig. 2B). Tumor cells, tolerogenic DCs, TAMs, and/or myeloid-derived suppressor cells (MDSCs) can express indoleamine-2,3-dioxygenase (IDO). This enzyme reduces tryptophan availability and generates the immunoregulatory metabolite kynurenine, which can promote T_reg_ cell differentiation and support the stability of activated T_reg_ cells in the TME [117, 118]. Mechanistically, kynurenine directly promotes the nuclear localization and activation of aryl hydrocarbon receptor (AHR), a transcription factor that is critical for T_reg_ cell differentiation in the gut [119]. Inhibiting kynurenine–AHR interactions is sufficient to disrupt the IDO-associated immunosuppressive axis between intratumoral T_reg_ cells and TAMs [120, 121]. Further, T_reg_ cells may directly induce IDO expression in DCs via CTLA4–CD80 interactions [122], thereby fostering a positive feedback loop for immunosuppression in the TME. In addition, arginine is a critical amino acid to license mTOR signaling during T_reg_ cell activation [65]. Interestingly, intratumoral T_reg_ cells show increased expression of arginase-2 that dampens mTOR signaling, thereby maintaining T_reg_ cell stability and functionality in tumors [123].

T_reg_ cells also influence the function of neighboring immune cells by altering the type and availability of various nutrient or energy signals. For example, intratumoral T_reg_ cells indirectly support SREBP function and lipid metabolism of immunosuppressive TAMs via suppression of CD8^+^ T cells [124], thereby driving an immunosuppressive feedforward loop in the TME. Another important example is T_reg_ cell-dependent modulation of extracellular ATP, which signals through purinergic receptors to inhibit T_reg_ cell function [125]. To circumvent this inhibitory effect, T_reg_ cells have elevated expression of CD39 and CD73, which hydrolyze extracellular ATP to AMP and AMP to adenosine, respectively [50, 126]. Of note, adenosine activates A_2A_ receptor signaling in effector T cells and APCs, which dampens the anti-tumor function of those cells [51, 126]. Further, mice lacking either CD39 or CD73 show enhanced anti-tumor immunity [127, 128]. Interestingly, CD39 and CD73 remain enzymatically active on T_reg_ cells after undergoing cellular apoptosis, thereby providing a mechanism for continued immunosuppression in tumors upon T_reg_ cell death [51]. As exhausted CD8^+^ T cells may upregulate CD39 expression and promote immunosuppression in tumors [129], this pathway is likely an important therapeutic target as discussed in more detail below. Collectively, these studies demonstrate that T_reg_ cells are poised for metabolic adaptation within the TME.

## Immune checkpoint blockade and T_reg_ cell metabolism

**Fig. 3:**
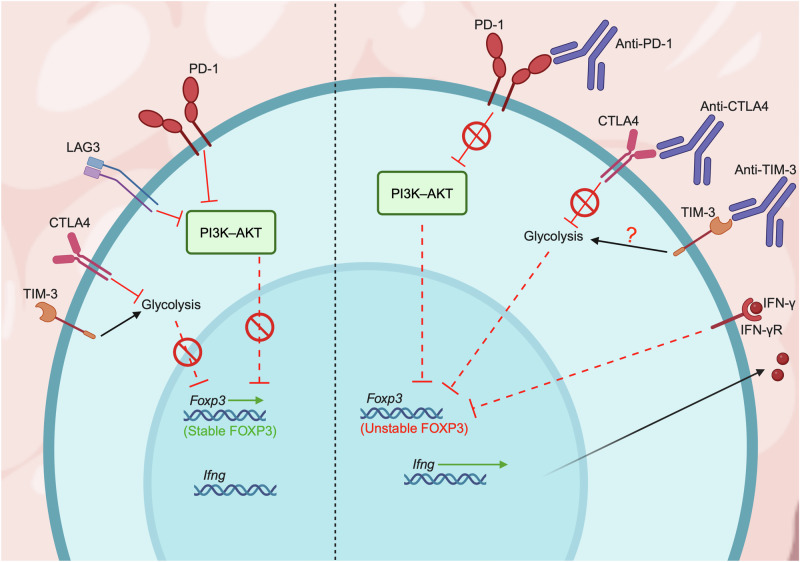
Immune checkpoint blockade and T_reg_ cell metabolism. Immune checkpoint molecules such as PD-1, LAG3, and CTLA4 help to stabilize *Foxp3* expression by dampening PI3K–AKT signaling and glycolysis. TIM-3 expression is associated with enhanced glycolysis in T_reg_ cells. Deletion of PD-1 or blockade of CTLA4 signaling is associated with increased PI3K–AKT signaling and glycolysis pathways in T_reg_ cells, thereby reducing FOXP3 stability; this effect is associated with increased autocrine IFN-γ signaling, which further destabilizes T_reg_ cell identity.

Biologic therapies aimed at reinvigorating anti-tumor immune responses (e.g., anti-PD-1 and anti-PD-L1 antibodies) may also positively affect T_reg_ cells, and this phenomenon impairs the overall treatment efficacy. In this section, we discuss how ICB reshapes T_reg_ cell metabolism and function, based primarily on studies in mouse models of cancer (Fig. 3).

Inhibitory molecules such as PD-1, LAG3, and CTLA4 on T_reg_ cells serve important roles in suppressing the activation of nearby immune cells and represent perturbation targets to unleash anti-tumor immunity. Accordingly, though anti-PD-1 ICB targeted at CD8^+^ T cells has shown clinical efficacy, there remains a high rate of non-responder patients, and such effects may be partly attributed to increased function and accumulation of PD-1^+^ T_reg_ cells upon anti-PD-1 treatment [130, 131]. Further, the uptake of lactate by T_reg_ cells within highly glycolytic tumors directly promotes PD-1 expression, thereby facilitating an anti-PD-1 treatment-induced reinvigoration effect on T_reg_ cells associated with treatment failure [80]. In turn, PD-1 signaling inhibits PI3K–AKT signaling and preserves the metabolic fitness of T_reg_ cells [130, 132, 133]. Of note, PD-1 is a complex regulator of T_reg_ cell biology. PD-1 restrains T_reg_ cell activation, with PD-1 targeting in T_reg_ cells improving their immunosuppressive function in mouse models of autoimmunity owing to the accumulation of highly activated eT_reg_ cells [131, 133]. Additionally, deficiency of LKB1, a crucial regulator of metabolic homeostasis, results in pronounced loss of T_reg_ cell function associated with aberrant upregulation of PD-1 and other immunoregulatory molecules [134], suggesting that PD-1 inhibits T_reg_ cell function under homeostasis. In contrast, PD-1 deletion specifically in T_reg_ cells reduces their stability or promotes fragility to impede tumor growth [92, 132]. Nonetheless, anti-PD-1 blockade often promotes the accumulation of highly suppressive eT_reg_ cells in the TME, thereby limiting ICB efficacy and promoting tumor growth [43, 130, 131]. Although intrinsic PD-1 signaling in T_reg_ cells may be involved as described above, these effects are also attributed to effects of anti-PD-1 blockade at increasing intratumoral IL-2 production by CD8^+^ T cells, which cooperates with ICOS signals to promote eT_reg_ cell expansion in tumors as noted above [43]. Thus, combination therapy approaches to co-target PD-1 with other T_reg_ cell-targeted approaches will likely be beneficial to counteract the effects on improving T_reg_ cell function under conditions of PD-1 blockade.

More recently, LAG3 was shown to inhibit PI3K–AKT and MYC signaling in T_reg_ cells [37], suggesting that the shortcomings of different ICB targets may be mechanistically linked to metabolic invigoration of T_reg_ cells in tumors, though this has not yet been clinically established. Of note, while PTEN deletion in T_reg_ cells leads to the development of autoimmunity [67, 68], PTEN targeting also reduces T_reg_ cell suppressive function in the TME [118]. Interestingly, T_reg_ cells rely upon the PI3K isoform p110δ, whereas p110δ, p110α, and p110β isoforms are functionally redundant in conventional T cells, indicating that pharmacological or genetic perturbation of p110δ may largely affect T_reg_ cells over conventional T cells. Indeed, mice with T_reg_ cell-specific inactivation of p110δ or those treated with a p110δ-specific inhibitor show increased anti-tumor immunity [135, 136], further illustrating the metabolic “Goldilocks” effect for T_reg_ cell function [69]. Further, intermittent dosing with the p110δ inhibitor AMG319 circumvents systemic immune-related adverse events while preserving anti-tumor immune phenotypes in humans and mice [137]. Direct pharmacological activation of AKT also boosts anti-tumor immunity by converting T_reg_ cells into T_H_1-like, IFN-γ-producing cells, thereby destabilizing FOXP3 expression [138]. Though T_H_1-like T_reg_ cells have important immunosuppressive functions, especially in the contexts of infection or autoimmunity [139], the conversion of intratumoral T_reg_ cells into T_H_1-like T_reg_ cells (and autocrine IFN-γ signaling) is mechanistically critical for anti-PD-1 ICB efficacy [140]. Additionally, scRNA-seq analysis in human patients has found that T_H_1-like T_reg_ cells are enriched in tumors that are responsive to anti-PD-1 ICB [141], and PD-1 deletion specifically in T_reg_ cells causes intratumoral T_reg_ cells to produce IFN-γ [92]. Together, these findings suggest that therapeutic manipulation of PI3K–AKT signaling in intratumoral T_reg_ cells can improve anti-PD-1 ICB efficacy via its direct causal link to IFN-γ-induced T_reg_ cell fragility.

As discussed above, T_reg_ cells take advantage of the lactate-rich TME by utilizing this by-product for metabolic and functional support [80–82]. However, glycolytic activity is markedly variable, especially between tumors originating in different tissues [142] (e.g., lung versus liver), and these differences in lactate availability may affect T_reg_ cell phenotypes that are relevant to ICB. Specifically, lactate promotes PD-1 expression on intratumoral T_reg_ cells via increased transcription factor NFAT1 activity; thus, T_reg_ cells from more glycolytic tumors show increased PD-1 expression and are resistant to anti-PD-1 ICB [80]. In addition to blocking co-stimulatory molecules, CTLA4-mediated interactions between T_reg_ cells and adjacent immune cells can dampen immune cell glycolysis. Thus, in tumors with low glycolytic activity (i.e., higher glucose availability for infiltrating immune cells), anti-CTLA4 blockade improves anti-tumor immune responses by permitting increased glycolysis; this is beneficial for CD8^+^ T cell activation and effector function but is detrimental to T_reg_ cell stability [86]. Of note, human T_reg_ cells require glycolysis to support *FOXP3* expression, proliferation, and suppressive function [76, 143], suggesting that anti-CTLA4 or other ICB therapies may impart discrete mechanistic effects in human and mouse T_reg_ cells. Likewise, TIM-3 is another clinically relevant inhibitory molecule that is highly expressed on intratumoral T_reg_ cells [144, 145], but its expression is instead associated with enhanced glycolysis and dampened OXPHOS metabolic phenotypes [146]. Further, TIM-3^+^ T_reg_ cells have enhanced suppressive function and increased expression of IL-10, and mice with enforced expression of TIM-3 on T_reg_ cells show impaired anti-tumor immunity and increased tumor growth [146]. This positive association of suppressive function and glycolytic metabolism suggests a complex signaling network downstream of inhibitory molecules that direct T_reg_ cell function in tumors; thus, more work is needed to clarify the discrete metabolic effects of inhibitory molecules, especially for those effects that may be species-dependent (i.e., human versus mouse). Taken together, these studies indicate that lowering metabolic competition may prime the TME for efficacious ICB effects by shifting the conditions favoring T_reg_ cell function to those favoring CD8^+^ T cells.

As previously stated, increased lipid metabolism is another important metabolic adaptation of intratumoral T_reg_ cells [92–94], and lipid synthesis pathways are critical for PD-1 expression (but not other T_reg_ cell-activation-associated molecules) specifically in intratumoral T_reg_ cells [92]. Accordingly, inhibition of lipid synthesis in T_reg_ cells leads to decreased PD-1 expression concomitant with enhanced PI3K–AKT signaling and IFN-γ production, and these effects sensitize B16F10 melanoma to anti-PD-1 treatment [92]. In radiotherapy-treated glioblastoma, anti-PD-1 treatment after receiving radiotherapy leads to a selective increase of highly suppressive CD103^+^ T_reg_ cells with a selective enrichment of lipid metabolism signatures [147]. Further, T_reg_ cell depletion following radiotherapy reverses the effects of anti-PD-1 treatment, which induces anti-tumor immune responses against ICB-resistant glioblastoma [147]. Together, these studies suggest that targeting lipid metabolism in intratumoral T_reg_ cells may be critical for improving ICB efficacy.

Aside from TME-derived molecules, host factors may also influence anti-tumor immunity by acting upon T_reg_ cells. Obesity is a systemic metabolic disorder associated with excessive adipose tissue and increased incidence and progression of cancers [148], and it is increasingly understood that obesity is accompanied by dysregulated immunity [149, 150]. PPAR-γ^+^ T_reg_ cells maintain immune and metabolic homeostasis within visceral adipose tissue (VAT) [151]; however, VAT-resident T_reg_ cells are decreased in frequency in obesity, and this effect is coincident with increased pro-inflammatory responses [152, 153]. Despite an increase in baseline inflammation, obesity has a suppressive impact on anti-tumor immunity [154] that is possibly due to suboptimal anti-tumor immune surveillance [155]. Intriguingly, several studies highlight a positive correlation between obesity and responsiveness to ICB [155–157], termed the ‘obesity paradox’. One proposed mechanism for this phenomenon is increased tumor immunogenicity due to impaired CD8^+^ T cell function (prior to ICB), which imparts a functional advantage to those CD8^+^ T cells reinvigorated by ICB [155]. Nonetheless, the effects of obesity on T_reg_ cell metabolism and immunosuppressive function in the TME remain incompletely understood.

The microbiome is another factor with a proven influence on cancer development [158, 159] and immunotherapeutic efficacy [160–162]. In the intestines, T_reg_ cell differentiation and function are regulated by microbiota-derived metabolites such as short-chain fatty acids [163, 164] and secondary bile acids [165–167], which support T_reg_ cell mitochondrial fitness. The possible impact of microbiota-derived metabolites on T_reg_ cells in cancer is also emerging. Indeed, patients with elevated microbiota-derived short-chain fatty acids have increased proportions of T_reg_ cells and are more resistant to anti-CTLA4 treatment [168], suggesting that microbiome composition-associated effects on T_reg_ cells are clinically relevant and may be used as a prognostic marker or manipulated for ICB efficacy. Taken together, metabolism-associated host factors such as obesity and microbiome composition have clear consequences in cancer, and thus, future work should mechanistically dissect how T_reg_ cells are affected by these important aspects.

## The search for specificity in targeting intratumoral T_reg_ cells

**Fig. 4:**
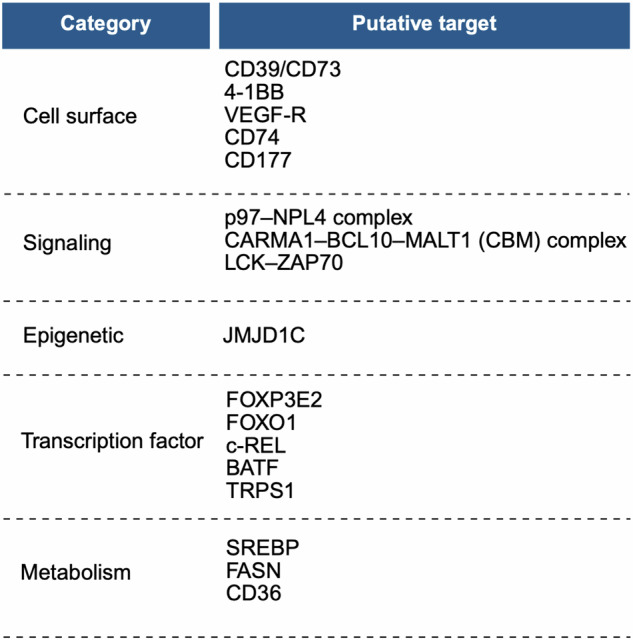
Putative therapeutic targeting of intratumoral T_reg_ cells. Various molecules and/or pathways may be exploited to specifically target T_reg_ cells in the TME. Those targets expressed on the cell surface include the extracellular adenosine-producing enzymes CD39 and CD73, the T_reg_ cell activation marker 4-1BB, the receptor for VEGF (VEGF-R), the invariant chain of MHC-II (CD74), and the neutrophil-associated marker CD177. Additionally, some therapeutic targets are associated with cell signaling pathways, including the ATPase p97 in complex with its co-factor NPL4 (p97–NPL4 complex), the CARMA1–BCL10–MALT1 (CBM) complex, and LCK–ZAP–70 signaling downstream of TCR activation. The histone demethylase JMJD1C serves as an epigenetic modifier target to disrupt intratumoral T_reg_ cells. Transcription factors whose disruption is associated with impaired intratumoral T_reg_ cell function include FOXP3E2, FOXO1, c-REL (subunit of the canonical NF-κB complex), BATF, and TRPS1. Finally, direct metabolic targets include SREBP and FASN, associated with de novo fatty acid synthesis, and the fatty acid transporter CD36.

T_reg_ cells are a major factor limiting the potential of immunotherapies. However, it is now understood that T_reg_ cell functions extend to homeostatic tissue maintenance (e.g., in the skin [169]) and wound repair (e.g., in damaged muscle tissue [170]), and adoptive T_reg_ cell therapies have been proposed for treatment of non-immune diseases [171]. Moreover, depletion of bulk T_reg_ cells may consequently induce conventional CD4^+^ T cells to express the immunosuppressive cytokine IL-10 [172], causing a paradoxical immunosuppressive barrier to anti-tumor immunity. Awareness of these and other possible adverse events associated with the indiscriminate inhibition of T_reg_ cells has resulted in much effort to identify molecular mechanisms utilized specifically by T_reg_ cells in tumors, as reviewed elsewhere [7, 8, 24, 171, 173]. In this section, we describe the potential therapeutic targets relevant to this notion, with a particular focus on metabolism-associated molecules that have shown promise as therapeutic targets (Fig. 4).

Given that intratumoral T_reg_ cells express CD39 and CD73 to mediate immunoregulatory effects [51, 125], this enzymatic pathway is of therapeutic interest. Indeed, antibody-mediated neutralization of CD39 and/or CD73 increases effector T cell activation and reduces tumor growth [127, 128, 174, 175]. Further, CD73 blockade synergizes with anti-PD-1 therapy in murine models of pancreatic cancer [175]. The expression of the T_reg_ cell-activation-associated molecule 4-1BB is also linked to various types of cancers [176], highlighting its potential as a therapeutic target. Mice with autophagy-defective T_reg_ cells also show enhanced anti-tumor immunity and control of tumor growth [66, 177, 178], suggesting that this may be a viable therapeutic target. Accordingly, tumor cell-intrinsic upregulation of autophagy is a common mechanism for immune evasion via decreased MHC-I expression on the cell surface [179], further indicating that targeting of this process may improve disease outcomes. Indeed, genetic or pharmacological inhibition of autophagy enhances anti-tumor immunity and sensitizes previously non-responding tumors to ICB [179]. Moreover, inhibition of tumor cell-intrinsic autophagy reduces tumor growth, with such effects partly related to reduced intratumoral T_reg_ cell accumulation [180]. Thus, tumor cell–T_reg_ cell metabolic interplay via autophagy may shape cancer therapeutic outcomes.

Other studies have identified the expression of unique genes in intratumoral T_reg_ cells from various cancer types. Developing tumors produce angiogenic signals such as VEGF, and T_reg_ cells expressing VEGF receptor (VEGF-R) infiltrate tumors and proliferate in response to tumor-derived VEGF [109, 110, 181]. Antibody-mediated blockade of VEGF–VEGFR signaling limits intratumoral T_reg_ cell accumulation and sensitizes ICB-resistant tumors to anti-PD-1 treatment [181, 182]. Additionally, intratumoral T_reg_ cells in humans overexpress CD74 (the invariant chain of MHC-II), and CD74-deficient T_reg_ cells show decreased activation and accumulation in tumors [183]. The neutrophil-associated marker CD177 is also enriched on T_reg_ cells in renal clear cell carcinoma patients with poor prognoses [184]; antibody-mediated blockade or T_reg_ cell-specific deletion of CD177 decreases T_reg_ cell accumulation in tumors and improves tumor control [184]. Finally, a previously known anti-tumor drug target was recently shown to have an unexpected role in intratumoral T_reg_ cells; specifically, the ATPase p97 in complex with co-factor NPL4 functions to dampen STAT3 signaling and preserve T_reg_ cell identity and function in tumors [185]. Future studies can explore whether and how these molecules interplay with metabolism to shape T_reg_ cell stability and function.

TCR-related signaling is another area of interest to target intratumoral T_reg_ cells. For example, disruption of the CARMA1–BCL10–MALT1 (CBM) complex via deletion of one *Carma1* allele induces an IFN-γ^+^ fragile T_reg_ cell phenotype, which improves overall tumor control and sensitizes tumors to ICB [186], thereby demonstrating a mechanism to modify TCR–PKC signaling and “rewire” T_reg_ cell function. Interestingly, a small-molecule tyrosine kinase inhibitor (imatinib) used to treat chronic myelogenous leukemia has off-target inhibitory effects on LCK and ZAP-70, which are downstream of TCR activation. Imatinib treatment promotes a selective loss of activated T_reg_ cells, but not tumor antigen-specific CD8^+^ T cells, due to the relatively lower levels of tonic LCK signaling in activated T_reg_ cells, thus rendering them more susceptible to TCR deprivation-induced apoptosis [187].

FOXP3 proteins include several splicing variants associated with differential impacts on cellular metabolism and suppressive function [143], suggesting that these variants could be involved in regulating intratumoral T_reg_ cells. Indeed, the exon 2 splicing variant of FOXP3 (FOXP3E2) is promoted by CXCL12–CXCR4 signaling in the TME and is associated with poor prognosis in patients with breast cancer [188]. Further, FOXP3E2^+^ T_reg_ cells show relative impairments in glycolysis compared to T_reg_ cells expressing full-length FOXP3 [143, 188], further supporting the notion of enhanced metabolic adaptation of T_reg_ cells within the TME. Besides FOXP3, additional transcription factors regulate and coordinate T_reg_ cellular functions with context- and/or tissue-dependent specificity, including the support of T_reg_ cell identity [189]. Because their perturbance can result in many downstream effects being altered, including metabolic effects [54, 58, 92, 190, 191], the search for putative transcription factor targets in intratumoral T_reg_ cells has been a primary area of focus. One of the first candidates to be described is the AKT signaling target FOXO1, which plays critical roles in T_reg_ cell activation, metabolism, and survival [77, 192]. Activated T_reg_ cells dampen FOXO1 signaling, which allows for expression of trafficking molecules and accumulation in tissues such as tumors [193]. Importantly, bi-allelic expression of a constitutively active FOXO1 mutant results in systemic functional defects in T_reg_ cells and autoimmune disease; however, mono-allelic expression of this constitutively active mutant reduces T_reg_ cell accumulation specifically in tumors, thus enhancing anti-tumor immunity without systemic autoimmune effects [193]. Additionally, c-REL, a subunit of the canonical complex of the transcription factor NF-κB, is critical for the differentiation of activated T_reg_ cells commonly found in tumors [194, 195]. Accordingly, c-REL inhibition in T_reg_ cells reduces their function in tumors and potentiates the effects of anti-PD-1 ICB [196]. BATF is also a major coordinator of T_reg_ cell activation and function in tumors [197, 198], although the function of BATF also extends to T_reg_ cell functions in multiple non-lymphoid tissues [199, 200]. A more recent study used pooled CRISPR–Cas9 screening combined with the chimeric immune editing (CHIME) model to investigate putative intratumoral T_reg_ cell master transcriptional regulators nominated from analysis of primary human patient samples [201]; this study described TRPS1 as a “master” transcriptional regulator of intratumoral T_reg_ cells versus peripheral T_reg_ cells. As such, genetic or pharmacological inhibition of TRPS1 specifically depletes intratumoral T_reg_ cells, inhibits tumor growth, and increases the efficacy of anti-PD-1 ICB [201]. Similar to context-specific transcription factors, T_reg_ cells in different tissues are epigenetically heterogeneous [16, 202], suggesting that epigenetic modulators may also be targetable in intratumoral T_reg_ cells. Indeed, the histone demethylase JMJD1C limits AKT signaling-induced IFN-γ production in intratumoral T_reg_ cells, and inhibiting JMJD1C function in T_reg_ cells selectively impairs intratumoral, and not peripheral, T_reg_ cell function [203].

Advances in biomedical engineering and synthetic biology have allowed for exciting new strategies in anti-tumor immunotherapy. For example, because of the requirement for IL-2 signaling for successful rejuvenation of intratumoral CD8^+^ T cells, one study engineered anti-PD-1 paired with a low-affinity IL-2 molecule, which showed reduced effects on T_reg_ cells but stronger and more specific effects on CD8^+^ T cells [204]. Another biomedically engineered antibody with bi-specific targeting of CD25 and TIGIT promotes T_reg_ cell depletion specifically in tumors [205]. Moreover, rather than directly inhibiting the molecular and metabolic pathways that confer functional advantages to T_reg_ cells in tumors, it may be feasible to imbue those features into CD8^+^ T cells used in ACT. As proof-of-principle, CD8^+^ T cells with enforced expression of FOXP3 gain T_reg_ cell-associated metabolic adaptations in tumors, including enhanced lipid metabolism in nutrient-limited conditions [206]. When used for ACT, these FOXP3^+^CD8^+^ T cells showed improved recruitment and cytotoxicity in tumors [206]. Similar immunometabolic effects are observed in intratumoral CD8^+^ T cells upon treatment with a bioengineered IL-10–Fc fusion protein [207] and in CAR T cells with enforced expression of IL-10 [208]. In summary, targeting T_reg_ cell accumulation or immunosuppressive functions holds tremendous promise for cancer immunotherapy, and we are only beginning to understand how targeting T_reg_ cell metabolism contributes to these therapeutic benefits.

## Conclusions

T_reg_ cells are a critical component of the immune system in distinguishing self from non-self and in minimizing the deleterious effects of inflammation. However, such functions of T_reg_ cells underlie why many tumors are non-responsive to immunotherapies, and thus, a greater understanding is needed for how T_reg_ cells mechanistically thrive within the TME. Understanding these context-dependent mechanisms is especially important, due to adverse effects of systemic T_reg_ cell depletion or functional blockade [171, 209]. There is increasing evidence that T_reg_ cells metabolically adapt to the harsh TME, and these unique survival mechanisms may be the key to specifically targeting those cells and also informing the future design of CD8^+^ ACT [206]. In this review, we have discussed how metabolic factors shape T_reg_ cell function against tumors and metabolism-related targets that affect intratumoral T_reg_ cell biology of various cancers. These important findings have translational potential, either as new therapies or for use to bolster existing therapies in combination.

Cancer is a highly heterogeneous condition with starkly different inter-tissue and intra-tissue phenotypes that are likely to impact T_reg_ cell metabolism, heterogeneity, and function. Our knowledge of the metabolic adaptations of T_reg_ cells in tumors has advanced in recent years. However, there is still much to be learned about how T_reg_ cells seemingly thrive in this harsh environment, and also how these findings in pre-clinical models translate into the clinic. Recent advances in single-cell and/or spatial metabolomics [1, 210] combined with assays to uncover transporters, sensors, or signaling transducers of nutrients or metabolites [64] will advance our understanding of how metabolic adaptation or signaling regulates intratumoral T_reg_ cell function. In this regard, flow cytometry-based approaches such as SCENITH [211] could be used to explore metabolic profiles at the single-cell level. Additionally, in vivo tracing of stable isotopes and imaging analysis by positron emission tomography may illuminate the spatial regulation of T_reg_ cell metabolism in the TME as previously demonstrated in other cell types [212, 213], thereby enhancing our knowledge of nutrient utilization, metabolic reprogramming, and metabolic signaling that shapes intratumoral T_reg_ cell function.

The inhibition of intratumoral T_reg_ cells can enhance tumor sensitization to ICB, supporting the notion that combination therapy to neutralize T_reg_ cell functions in tumors may unleash the full potential of ICB. However, we have limited understanding of the specific pathways that shape intratumoral T_reg_ cell fitness versus their counterparts in healthy lymphoid and non-lymphoid tissues. To this end, powerful screening technologies such as CRISPR–Cas9 have revolutionized our ability to discover and test previously unknown regulators of T cell function [214]. Though genome-wide CRISPR screening can identify targets to improve CD8^+^ T cell-mediated ACT [215, 216], the use of CRISPR-based screening in T_reg_ cells has been less commonly applied. Modified CRISPR-based strategies such as CHIME [201, 217, 218] may permit screening for functional regulators of T_reg_ cells, including novel targets that convey functional advantages to T_reg_ cells in tumors. Further, the application of single-cell CRISPR screening technology [219] to T_reg_ cells will potentially illuminate unknown gene regulatory networks and T_reg_ cell heterogeneity to exploit for therapeutic benefit.
